# Hypoplastic superficial brachioradial artery coexisting with atypical formation of the median and musculocutaneous nerves: a rare combination of unusual topographical relationships

**DOI:** 10.1007/s00276-019-02183-1

**Published:** 2019-01-17

**Authors:** Robert Haładaj, Grzegorz Wysiadecki, Michał Polguj, Mirosław Topol

**Affiliations:** 1 Department of Normal and Clinical Anatomy, Interfaculty Chair of Anatomy and Histology, Medical University of Lodz, ul. Żeligowskiego 7/9, 90-752 Łódź, Poland; 2 Department of Angiology, Interfaculty Chair of Anatomy and Histology, Medical University of Lodz, ul. Żeligowskiego 7/9, Łódź, Poland

**Keywords:** Anatomic variation, Arteries, Brachial plexus, Median nerve, Musculocutaneous nerve, Radial artery

## Abstract

The use of the term “brachioradial artery” was introduced for the high origin of the radial artery. Although the prevalence of the brachioradial artery reported by different authors varies from 4.67 to 15.6%, the presence of the hypoplastic brachial segment of the brachioradial artery is rare with an occurrence rate of 0.83%. Moreover, in just 0.6% of cases the loop of the median nerve may be placed near half of the length of the brachial artery, as in the case described in our report. A comprehensive understanding of anatomical variations of neurovascular structures in the upper limb is of great clinical significance. The presented case report illustrates a rare manifestation of persistent primitive developmental relationships in the arterial pattern of the upper limb (persistent, hypoplastic brachial segment of the superficial brachioradial artery), coexisting with atypical formation of the median and musculocutaneous nerves. Anatomical variations of vessels and nerves may coexist which should be taken into account when performing vascular, reconstructive or orthopedic surgery.

## Introduction

The radial artery demonstrates a high variability in regard to its origin, course, diverse arrangements of radial recurrent artery and its contribution to vascularization of the hand [3, 7, 12–14, 18]. Rodríguez-Niedenführ et al. [14] proposed the term “brachioradial artery” to be used for the high origin of the radial artery. In such a case, the accessory artery is observed in the medial bicipital sulcus, running superficially to the median nerve. Although the prevalence of the brachioradial artery reported by different authors varies from 4.67 to 15.6% [3, 7, 12, 14], the presence of the hypoplastic brachial segment of the brachioradial artery is rare with an occurrence rate from 0.67% [7] to 0.83% [3].

A comprehensive understanding of the anatomical variations of neurovascular structures in the upper limb is of great clinical significance [2, 9–11]. The brachioradial artery may form an anastomosis with the brachial artery proper in the cubital fossa (so-called “cubital anastomosis”, also referred to as “cubital crossover” or “cubital connection”) [3, 7, 12–14]. Such anastomosis is observed in 17.8–54.55% of cases and may be of great importance during radial artery catheterization [3, 7, 10, 11]. In the clinical context, it is also important to know possible anatomical variations of the median nerve and the musculocutaneous nerve [5, 8, 16]. In some cases, the coexistence of anatomical variations of the nerves and vessels can even be a source of conflict between individual structures, e.g., entrapment neuropathy of the median nerve due to the atypical (superficial to the nerve) course of the brachial artery [9].

The presented case report illustrates a rare manifestation of persistent primitive developmental relationships in the arterial pattern of the upper limb (i.e., persistent, hypoplastic brachial segment of the radial artery), coexisting with an atypical formation of the median and musculocutaneous nerves.

## Case description

The case described in this paper was an incidental finding during routine dissection of an isolated upper limb fixed in 10% formalin solution. The procedure was performed using microsurgical instruments at a magnification of 2.5× under a HEINE^®^ HR 2.5× High Resolution Binocular Loupe (HEINE Optotechnik GmbH & Co. KG, Herrsching, Germany). The measurements were taken with a Digimatic Calliper (Mitutoyo Corporation, Kawasaki-shi, Kanagawa, Japan).

**Fig. 1:**
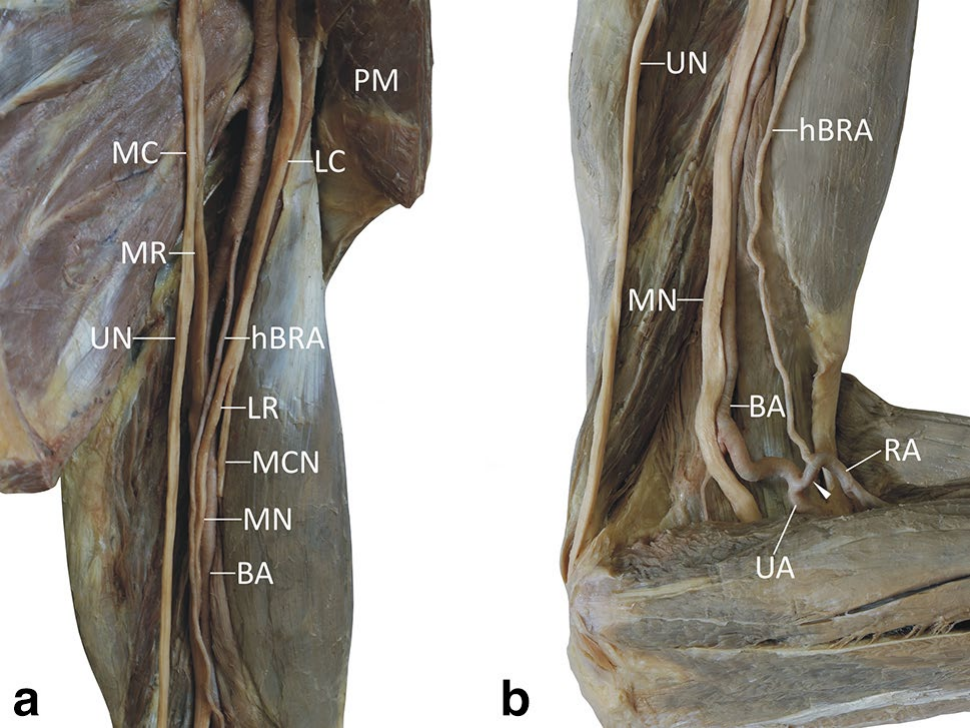
Hypoplastic superficial brachioradial artery branching off the proximal segment of the brachial artery. **a** Atypical anatomical relations within the medial bicipital sulcus. The superficial brachioradial artery runs superficial to atypical roots of the median nerve. **b** Anterior view to the cubital fossa. The cubital anastomosis between the brachioradial artery and brachial artery proper is exposed. Hypoplastic brachial segment of the superficial brachioradial artery is present. Within the forearm the radial artery (i.e., the forearm segment of the brachioradial artery) occupies the typical location. *AA* axillary artery, *BA* brachial artery, *hBRA* hypoplastic brachial segment of the superficial brachioradial artery, *LC* lateral cord of the brachial plexus, *LR* lateral root of the median nerve, *MC* medial cord of the brachial plexus, *MCN* musculocutaneous nerve, *MN* median nerve, *MR* medial root of the median nerve, *PM* pectoralis major muscle, *RA* radial artery, *UN* ulnar nerve, white arrowhead—cubital anastomosis

We observed a hypoplastic superficial brachioradial artery coexisting with variations of the median and musculocutaneous nerves. The superficial brachioradial artery (of 1.69 mm diameter) branched from the anterior aspect of the brachial artery (of 6.48 mm diameter) 8 mm below the level of inferior border of the pectoralis major muscle, 210 mm above the interepicondylar line of the humerus (Fig. 1a). The atypical artery along with accompanying veins emerged from between the atypical roots of the median nerve. The lateral root of the median nerve and the musculocutaneous nerve were fused on their initial course along the upper third of the arm. The careful dissection (the specimen of the brachial plexus was subsequently harvested and immersed in 10% acetic acid solution for 2 weeks to facilitate removal of the epineural sheath [4]) revealed that both these structures were surrounded by a common epineurium and could be separated only by the limited distance of approximately 80 mm. Moreover, the loop (the fork) of the median nerve was placed below the level of the axillary artery, at the brachial artery in midpoint of the arm (Fig. 1a). In the described case, the musculocutaneous nerve, due to its atypical formation, did not pierce the coracobrachialis muscle. The coracobrachialis muscle was one-headed (Fig. 3b). Single muscular branches to the coracobrachialis and biceps brachii muscles arose proximally to the side where the lateral root of the median nerve originated (Fig. 3b). The muscular branch to the brachialis muscle arose below the side of branching of the lateral root of the median nerve, directly from the musculocutaneous nerve. The lateral and medial roots of the median nerve formed the median nerve within midpoint of the arm (135 mm above the interepicondylar line of the humerus and 85 mm below the inferior border of the pectoralis major muscle; Fig. 1a). The further course of both the median and musculocutaneous nerves was normal. The musculocutaneous nerve continued as the lateral cutaneous nerve of forearm.

Within the proximal third of the medial bicipital sulcus, the hypoplastic brachial segment of the superficial brachioradial artery took a superficial course to both the roots of the median nerve (Fig. 1). On its further course along the arm, the artery gave one branch (of 0.57 mm diameter) to the biceps brachii muscle and, at some distance below, crossed the median nerve from above (72 mm above the interepicondylar line of the humerus). Within the medial bicipital sulcus, the brachial artery proper demonstrated normal course, deep to both atypical roots of the median nerve (in the proximal half) and deep to the median nerve (in the distal half). Within the anterior cubital region, the superficial brachial artery ran deep both to the superficial veins and deep fascia, but superficially to muscles, nerves, and other arteries. Within the cubital fossa the hypoplastic superficial brachioradial artery formed a strong cubital anastomosis (of 3.23 mm diameter) with the brachial artery proper (Fig. 1b). The observed anastomosis was characterized by a tortuous course. In the case described in this report, the cubital anastomosis branched off the brachial artery (of 4.94 mm diameter at the level of the cubital fossa) 14 mm below the interepicondylar line of the humerus. The anastomosis was located anterior to the distal tendon of the biceps brachii muscle. Since the anastomosis was characterized by larger diameter than the brachial part (segment) of the superficial brachioradial artery, it was classified as the “dominant type of the cubital anastomosis” (Fig. 1b). In the observed dominant type of the cubital anastomosis, the diameter of the superficial brachioradial artery just below the anastomosis increased significantly by 98.8% (from 1.63 to 3.24 mm). A single recurrent radial artery of 2.07 mm diameter arose from the superficial brachioradial artery 28 mm below the interepicondylar line of the humerus.

On its further course, the superficial brachioradial artery ran superficially along the border of the brachioradialis muscle and was covered only by the antebrachial fascia. Then it turned back at the wrist, behind the tendons of the abductor pollicis longus and extensor pollicis brevis muscles. The diameters of superficial brachioradial and ulnar arteries measured at the level of the wrist were 2.99 mm and 3.29 mm, respectively. The superficial palmar arch was classified as an incomplete ulnar arch, in which the superficial palmar branch of the radial artery did not contribute to formation of the arch. No other anatomical variations were observed on the examined specimen.

## Discussion

The process of the development of limb arteries has been the subject of a lot of research and controversy. Classical theories of development of upper limb arteries assumed that gradual sprouting of the arterial trunks takes place from a primordial axial artery [15]. In turn, Rodríguez-Baeza et al. [12] proposed a model based on the assumption that during normal morphogenesis, the upper limb arteries are formed by the union of both superficial and deep pathways, implying that the superficial brachial artery is a consistent embryonic vessel. On its course, the superficial brachial artery anastomoses with deep pathways. The anastomosis located in the cubital fossa plays a crucial role, giving rise to the typical origin of the radial artery. In most cases the pre-anastomotic segment of the superficial brachial artery regresses during arterial development [12]. The anatomic variation in which hypoplastic segment of brachioradial artery persists in the arm may be a confirmation of this direction of changes occurring at the stage of formation of the main arterial trunks of the upper limb. Recent models of the development of the upper limb arteries [13, 14, 17] assume that the definitive arterial pattern of the upper limb is formed as a result of capillary remodeling and subsequent differentiation of main arterial trunks and regression of the collateral pathways within the primitive capillary plexus.

The brachioradial artery may take an origin from the axillary or brachial artery [3, 7, 12, 14]. McCormack et al. [7] pointed out some important details about the topographical relations of the brachioradial artery. If the brachioradial artery branches off the brachial artery, its origin is most frequently located on the medial circumference of the brachial artery. Then, such a vessel runs medially and then anteriorly to the median nerve. In contrast, in the case described in this paper the superficial brachioradial artery arose from the anterior aspect of the brachial artery.

**Fig. 2:**
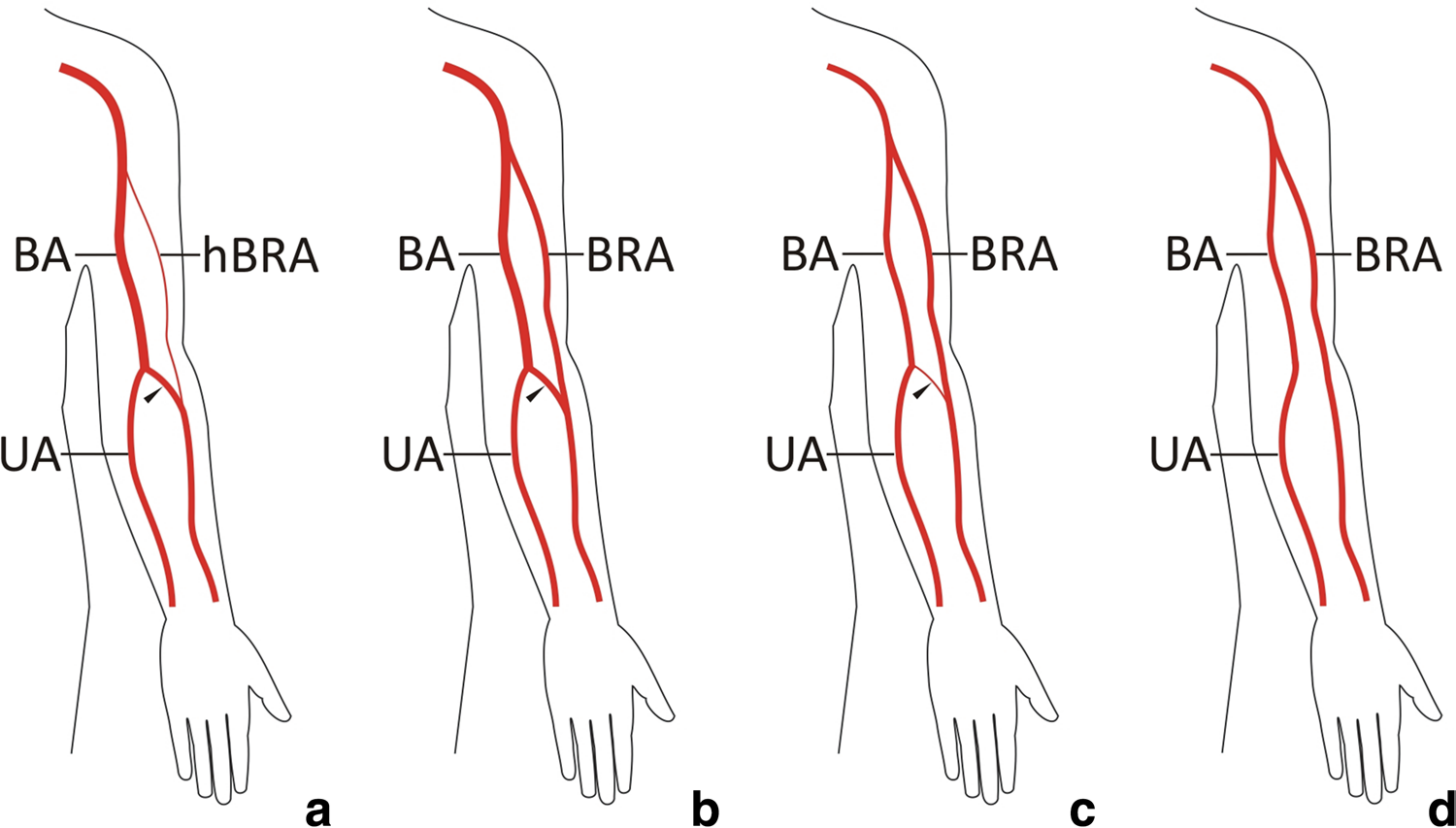
Anatomical variations of the cubital anastomosis between the brachioradial artery and brachial artery proper (based on Haładaj et al. [6]). **a** Variation described in our case report. Dominant type of the cubital anastomosis with hypoplastic brachial segment of the brachioradial artery. **b** Balanced type. This anastomosis type was characterized by a diameter similar to that of the brachioradial artery. **c** Minimal type. This anastomosis type was characterized by a diameter significantly smaller to that of the brachioradial artery. **d** Absence of anastomosis between the brachioradial artery and brachial artery proper within the cubital fossa. *BA* brachial artery, *BRA* brachioradial artery, *hBRA* hypoplastic pre-anastomotic segment of the brachioradial artery, *RA* radial artery, *UA* ulnar artery, black arrowhead—cubital anastomosis

A cubital anastomosis is an anastomotic artery running in a variable manner between the brachioradial artery and brachial artery proper (Fig. 2). Such a connection may be considered as a remnant of the anastomosis between the primitive axial and superficial brachial arteries at the level of the typical origin of the radial artery [12]. In our earlier study on anatomical variations of the brachioradial artery, performed on 120 upper limbs, we distinguished three types of the cubital anastomosis (cubital connection): dominant in 9.1% of specimens with brachioradial artery (Fig. 2a), balanced in 27.3% (Fig. 2b) and minimal in 18.2% (Fig. 2c) [3]. The cubital anastomosis was absent 45.4% of upper limbs with brachioradial artery (Fig. 2d) [3]. A very prominent anastomosis with a slender pre-anastomotic part of the radial artery of high origin (brachioradial artery) was observed by McCormack et al. [7] in 5 out of 750 limbs. Such a hypoplastic proximal (pre-anastomotic) segment of brachioradial artery was described by von Haller as *vasa aberrantia* as early as in 1753 [19].

Major anatomical variations of the upper limb arteries often coexist with the variations of radial recurrent arteries. According to Vazquez et al. [18], the radial recurrent artery takes its origin most frequently from the radial artery (64,8%), posterior radioulnar division (9%), anterior radioulnar division (5.4%), brachioradial artery—as in our case (7.8%), brachial artery (7.2%), ulno-interosseous trunk (2.7%), or even the interosseous trunk (0.3%). In turn, in the study of McCormack et al. [7], the radial recurrent artery arose from the cubital anastomosis in 10 out of 14 cases possessing this type of union. However, Rodríguez-Niedenführ et al. [14], found that when brachioradial artery was present, the radial recurrent artery took origin from it in 46% (as in our case), from the “normal” brachial artery in 34% and from the cubital anastomosis in 20%.

Since the radial artery is often used in vascular, plastic, and reconstructive surgery and routinely used for puncture and cannulation, knowledge of its variations can be of great clinical significance. Transradial access can be hindered by the presence of an unusual origin and course of the vessel [11]. Recent studies have indicated that the presence of a high origin of the radial artery (brachioradial artery) is associated with a more tortuous course of this artery, which can increase the risk of failure of transradial catheterization [10].

**Fig. 3:**
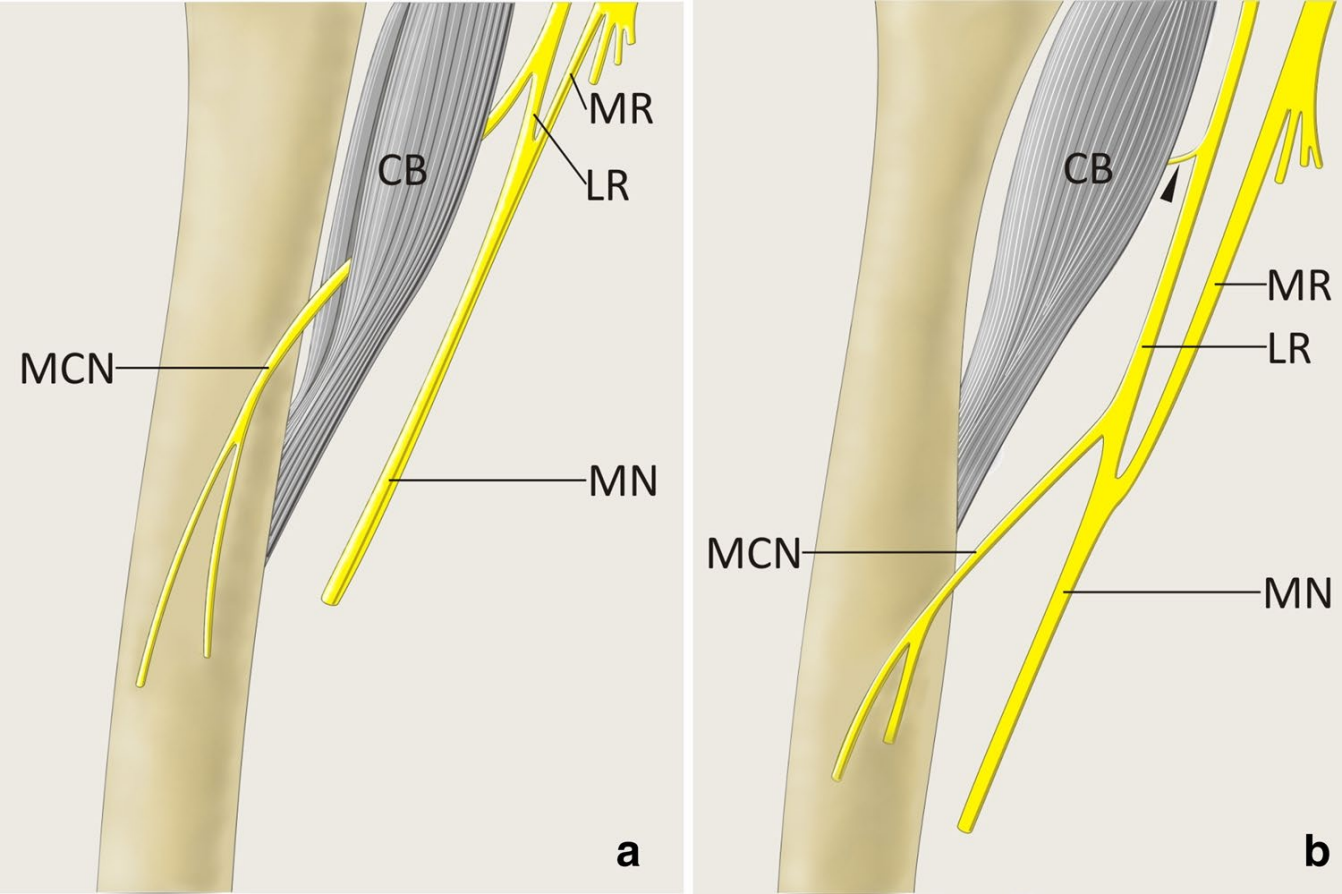
Two different anatomical variations of the musculocutaneous and median nerves. **a** Typical formation and course of the median (MN) and musculocutaneous (MCN) nerves. The median nerve originates in the axillary fossa from the medial (MR) and lateral (LR) root. The musculocutaneous nerve pierces the coracobrachialis muscle. **b** Variation described in this report. The musculocutaneous nerve is fused with the lateral root of the median nerve forming elongated lateral cord of the brachial plexus. Muscular branch (marked by black arrowhead) to the coracobrachialis muscle (CB) arises from the lateral cord of the brachial plexus. The coracobrachialis muscle is not pierced by any neural structure. The loop (the fork) of the median nerve is located on the arm

The case described in our report may also be considered a rare variant of the median and musculocutaneous nerves formation (Fig. 3b). The musculocutaneous nerve typically originates within the axillary fossa, from the lateral cord of the brachial plexus (Fig. 3a). The nerve descends into the arm passing through the coracobrachialis muscle (Fig. 3a). However, the musculocutaneous nerve does not pierce the coracobrachialis muscle in 1.8% of cases [2]. In some instances an atypical lateral cord of the brachial plexus may pierce the coracobrachialis muscle [6, 16]. The typical origin of the median nerve from the lateral and medial cords of the brachial plexus occurs in about 85% of specimens [6]. The loop (the fork) of the median nerve may be moved to the beginning or to the upper third of the brachial artery in about 8.4% of cases [2]. However, in just 0.6% of cases the loop of the median nerve is placed near half of the length of the brachial artery, as in the case described in our report [2].

The musculocutaneous and median nerves may be fused at some distance [5, 8]. The origin of the branch to the coracobrachialis muscle may be from the lateral cord of the brachial plexus [8, 16]. Absence of the musculocutaneous nerve with innervation of the coracobrachialis, biceps brachii, brachialis, and the skin of the anterolateral surface of the forearm by branches from the lateral cord of the brachial plexus was described by Nakatani et al. [8]. Sirico et al. [16] point out to the difficulty in comparing results between studies regarding the atypical morphology of the musculocutaneous nerve. According to those authors “sensory and motor branches are not consistently reported in the brachial plexus descriptions in dissection studies and there is no agreement regarding the actual sequence of these branches along the nerve” [16]. In our case the muscular branches to the brachial flexors were distributed in the order described by Hayashi et al. [5]; The most proximal was the branch to the coracobrachialis muscle, the second was the branch to the biceps brachii muscle and the most distal was the branch to the brachialis muscle. However, only the last branch arose directly from the musculocutaneous nerve.

Due to the fact that the musculocutaneous nerve is absent in lower vertebrates, the absence of this nerve in humans may be considered an ontological remnant during embryogenesis [6]. Anatomical studies of embryos suggest, that the musculocutaneous nerve is derived from the median nerve [6]. Thus, different anatomical variations of the formation of the median and musculocutaneous nerves may be examples of atypical pathways of the nerve growth. Moreover, an analysis of combined anatomical variations may contribute to a better understanding of the mechanisms that guide the morphogenesis of blood vessels and nerves. Carmeliet [1] points to the existence of common molecular signals and pathways during development of the blood vessels and nerves, which may explain the coexistence of anatomical variations of neurovascular structures. Combined anatomical variations of nerves and blood vessels, similar to those described in our report, may also constitute a potentially important clinical and surgical issue, especially during repair of the brachial plexus or its branches, as well as during surgical procedures performed within the upper limb [2, 5, 16].

## Conclusions

The anatomical variation of the superficial brachioradial artery, in which its hypoplastic brachial segment is persistent and the cubital anastomosis in place of the normal origin of this artery is preserved, may be considered as a trace of complex developmental relationships during formation of upper limb arteries. Variations of vessels and nerves may coexist and should be taken into account when performing vascular, reconstructive or orthopedic surgery.
